# A national‐scale model of linear features improves predictions of farmland biodiversity

**DOI:** 10.1111/1365-2664.12912

**Published:** 2017-05-07

**Authors:** Martin J. P. Sullivan, James W. Pearce‐Higgins, Stuart E. Newson, Paul Scholefield, Tom Brereton, Tom H. Oliver

**Affiliations:** ^1^ British Trust for Ornithology The Nunnery Thetford Norfolk IP24 2PU UK; ^2^ School of Geography University of Leeds Leeds LS2 9JT UK; ^3^ NERC Centre for Ecology & Hydrology Maclean Building Benson Lane Crowmarsh Gifford Wallingford Oxfordshire OX10 8BB UK; ^4^ Butterfly Conservation Manor Yard East Lulworth Wareham Dorset BH20 5QP UK; ^5^ School of Biological Sciences University of Reading Harborne Building Whiteknights Reading RG6 6AS UK

**Keywords:** abundance model, agriculture, bird, butterfly, GIS, Hedgerow, remote sensing, species distribution model

## Abstract

1. Modelling species distribution and abundance is important for many conservation applications, but it is typically performed using relatively coarse‐scale environmental variables such as the area of broad land‐cover types. Fine‐scale environmental data capturing the most biologically relevant variables have the potential to improve these models. For example, field studies have demonstrated the importance of linear features, such as hedgerows, for multiple taxa, but the absence of large‐scale datasets of their extent prevents their inclusion in large‐scale modelling studies.
2. We assessed whether a novel spatial dataset mapping linear and woody‐linear features across the UK improves the performance of abundance models of 18 bird and 24 butterfly species across 3723 and 1547 UK monitoring sites, respectively.
3. Although improvements in explanatory power were small, the inclusion of linear features data significantly improved model predictive performance for many species. For some species, the importance of linear features depended on landscape context, with greater importance in agricultural areas.
4. *Synthesis and applications*. This study demonstrates that a national‐scale model of the extent and distribution of linear features improves predictions of farmland biodiversity. The ability to model spatial variability in the role of linear features such as hedgerows will be important in targeting agri‐environment schemes to maximally deliver biodiversity benefits. Although this study focuses on farmland, data on the extent of different linear features are likely to improve species distribution and abundance models in a wide range of systems and also can potentially be used to assess habitat connectivity.

## Abstract 2

This study demonstrates that a national‐scale model of the extent and distribution of linear features improves predictions of farmland biodiversity. The ability to model spatial variability in the role of linear features such as hedgerows will be important in targeting agri‐environment schemes to maximally deliver biodiversity benefits. Although this study focuses on farmland, data on the extent of different linear features are likely to improve species distribution and abundance models in a wide range of systems and also can potentially be used to assess habitat connectivity.

## Introduction

Predictive modelling of species distributions and abundances is used in a wide range of conservation applications, such as predicting species responses to environmental change, identifying priority areas for conservation, and assessing the potential distribution of range expanding species (e.g. Araújo *et al*. 2004). A common approach is to model the occurrence or abundance of a species as a function of land cover (e.g. Hirzel *et al*. 2006). Such datasets are readily available and have proved useful for predictive modelling (Oliver *et al*. 2012). However, land‐cover classes do not always represent ecologically relevant habitat classifications. For example, habitat classes such as broadleaved woodland encompass stands of different ages, species composition and management; these differences will influence the suitability of stands for different species (e.g. Fuller *et al*. 2007). In addition, fine‐scale variation within land‐cover classes could influence their suitability for species, but are hard to capture as they are often smaller than the resolution of land‐cover maps [e.g. the UK land‐cover map (LCM) 2007 used 25‐m resolution imagery; Morton *et al*. 2011].

Advances in remote sensing and associated analysis using Geographic Information Systems (GIS) have allowed more detailed and potentially more biologically relevant classification of environmental variables, with promising applications for mapping biodiversity (Pettorelli *et al*. 2014). For example, remote‐sensing data have been used to model the distribution of primate species in tropical forests (Palminteri *et al*. 2012) and birds in temperate forests (Broughton *et al*. 2012), allowing the identification of suitable habitat within a single land‐cover class. Evaluations of new spatial datasets produced by these methods typically concentrate on their ability to accurately classify the physical environment (Chassereau, Bell & Torres 2011), but it is also important to test whether they improve models of species distributions and abundance, as this will influence their applied use (Borre *et al*. 2011).

Attempts to model biodiversity in agriculturally dominated landscapes have previously used land‐cover data relating to agricultural land use and extent of remnant semi‐natural habitats. However, a wealth of field studies have shown that linear features, such as hedgerows, banks and linear shelterbelts of mature trees, contribute greatly to the biodiversity value of farmland. For example, 64% of British butterfly species have been recorded using hedgerows (Dover & Sparks 2000), while hedgerow length is positively associated with small mammal biomass (Gelling, Macdonald & Mathews 2007) and the abundance of farmland bird species (Parish, Lakhani & Sparks 1995), although measures of hedgerow composition and structure are also important (e.g. Hinsley & Bellamy 2000). Non‐woody boundaries may also benefit biodiversity (Siriwardena, Cooke & Sutherland 2012); for example, grassy boundary features like banks provide resources for many butterfly (Sparks & Parish 1995) and bird (Vickery, Carter & Fuller 2002) species. As well as resource provision, linear features also influence microclimate (Dover, Sparks & Greatorex‐Davies 1997; Merckx *et al*. 2010) and potentially increase connectivity in agricultural landscapes (Davies & Pullin 2007).

Despite strong evidence for the importance of linear features from field studies, national‐scale assessments of their importance are lacking due to the absence of GIS data on their distribution. Here, we use a newly developed GIS dataset (Scholefield *et al*. 2016a) to relate the extent of linear and woody linear features to the abundance of 42 bird and butterfly species across Great Britain. We assessed whether incorporating linear features data improves the performance of models of bird and butterfly abundance and examine whether the importance of linear features varies between taxa. Such large‐scale modelling of biodiversity using linear features data has important implications for the management of landscapes to maintain high species abundances. For example, models of spatial variation in the importance of linear features could help target agri‐environment schemes promoting hedgerow planting. Although this study focuses on farmland biodiversity, linear features datasets similar to that used here have potential applications in a wide range of habitats.

## Materials and methods

### Species abundance data

We used data on bird and butterfly abundance in Great Britain from two national‐scale monitoring schemes, the Breeding Bird Survey (BBS) and United Kingdom Butterfly Monitoring Scheme (UKBMS). Both are described in detail elsewhere (BBS: Risely *et al*. (2013), UKBMS: Pollard & Yates (1993)). In brief, in the BBS volunteers walk two transects through a 1‐km square and record all birds seen and heard in three distance bands (0–25, 25–100, >100 m). Sites are visited early and late in the breeding season (April–June). We use the maximum count in either visit for analysis. We exclude flying birds, except for species that are likely to be recorded in flight while using habitat within a BBS square (of our study species these were aerial feeding hirundines, hovering kestrel *Falco tinnunculus* and displaying skylark *Alauda arvensis*). In the UKBMS, volunteers walk transects through each site (typically 2–4 km long) weekly from April to September and record all butterflies seen within a 5‐m distance band. Data collected between 2005 and 2009 were used in this study. The choice of this timeframe was motivated by the desire to maximise the sample of surveyed sites while ensuring abundance data were collected at a similar time to environmental data. Only sites surveyed for more than 1 year were included, giving a sample size of 3723 BBS and 1547 UKBMS sites. Although differences in sampling design meant that more BBS transects than UKBMS transects were located in agricultural areas, other landscape characteristics were comparable between survey schemes (Table S1, Supporting Information).

We restricted analysis to species that occur in agricultural areas and are, therefore, species for which linear features data are especially relevant. For birds, we selected the 18 species that were identified by Renwick *et al*. (2012) to preferentially use agricultural areas, while for butterflies we selected 24 species classified as wider countryside species (Table S2).

### Environmental data

Land‐cover data were obtained from LCM 2007. Land‐cover classes were aggregated in some instances (Table 1), and their proportion in 1‐km radius buffers around BBS square and UKBMS transect centroids was extracted in ArcMAP 10.0 (ESRI 2010). The 1‐km buffer was chosen as it encompasses all habitat found within a BBS square and has been found to be the scale that explains the most variation in population dynamics in UKBMS sites (Oliver *et al*. 2010).

**Table 1:** Environmental variables used in different model sets

Model term (if LCM classes have been aggregated constituent classes are in parenthesis)	Units	Explanatory variable set
Arable and horticulture	Proportion of buffer	Land cover (full, agriculture)
Improved grassland	Proportion of buffer	Land cover (full, agriculture)
Rough grassland	Proportion of buffer	Land cover (full, agriculture)
Calcareous grassland	Proportion of buffer	Land cover (full)
Other semi‐natural grassland (neutral grassland, acid grassland)	Proportion of buffer	Land cover (Full)
Broadleaved woodland	Proportion of buffer	Land cover (full)
Coniferous woodland	Proportion of buffer	Land cover (full)
Fen, marsh and swamp	Proportion of buffer	Land cover (full)
Heath and bog (heather, heather grassland, bog)	Proportion of buffer	Land cover (full)
Urban and suburban (urban, suburban)	Proportion of buffer	Land cover (full)
Freshwater	Proportion of buffer	Land cover (full)
Altitude	m above sea level/maximum altitude^a^	Land cover (full, agriculture)
Linear features length	m/100 000^a^	Linear features
Woody linear features length	m/100 000^a^	Linear features

All variables listed here were entered into models with linear and quadratic terms.

a Altitude and linear features length were both transformed this way so that their values ranged between 0 and 1, the same range as in variables from LCM2007.

Data on the extent of linear features were obtained from a recently developed model (Scholefield *et al*. 2016a; see for full details). We use two outputs from this model. First, we used the spatial framework from LCM 2007, which is based on the Ordnance Survey Mastermap with additional data on agricultural boundaries from government agricultural agencies (Morton *et al*. 2011). This gives the total extent of linear boundary features, irrespective of their type (i.e. hedgerow, bank). The second output is a model that classifies whether these boundary features are woody. This uses the difference between the canopy surface model and digital terrain model from the remote‐sensed NEXTMap dataset (5‐m resolution) to obtain the canopy height of each linear feature. Features were classed as woody (i.e. hedges and trees) if the mean canopy height was ≥0·58 m, minimum canopy height >−0·13 m and maximum canopy height ≤58 m. These thresholds were parameterised by minimising the difference between predicted and observed woody linear features extent from Countryside Survey 2007 (Scholefield *et al*. 2016a). Countryside Survey data come a stratified random sample of 591 1‐km squares designed to give a representative sample of Great Britain. This model predicts landscape level woody linear features extent (i.e. woody linear features length within ITE land‐class) with *R* ^2^ = 0·98 and correctly classifies 58–66% of individual features (Scholefield *et al*. 2016a). Because the model is limited to the 5‐m resolution of NEXTMap, it was not possible to obtain measures of woody linear features quality (e.g. whether a hedgerow has gaps or hedgerow width). Linear features in uplands (altitude >450 m), urban areas (>10% urban) or forests are not predicted by this method. Therefore, we excluded them from further analysis. The total length of linear features in each 1‐km buffer was calculated as the sum of the length of all linear features within the buffer. Both measures of linear features length were positively correlated (*r *= 0·46).

### Statistical analysis

We modelled bird and butterfly abundance at each site in each year as a function of environmental variables using generalised linear mixed models with a Poisson error term. We used an observation‐level random effect to account for overdispersion (Elston *et al*. 2001). This effectively assumes that data largely result from a Poisson process, but with additional normally distributed variation modelled by the observation‐level random effect. Not all sites were monitored in all years, so to account for year‐to‐year variation in abundance, we fitted year as a fixed effect, with site (i.e. BBS or UKBMS transect identity) as a random effect to account for the expected correlation between abundances at the same site in different years. We also expected sites close to each other to be spatially autocorrelated, so we used the 50‐km British Ordnance Survey grid square containing the BBS or BMS transect as a random effect to account for this. For birds, we used distance sampling to account for variation in detectability among habitats and visits. For each species, we fitted half‐normal detection functions to counts in each bounded distance band (<25 m and 25–100 m) using the mrds package (Laake *et al*. 2013) in the program r (R Core Team 2013). Visit date (i.e. early or late) and habitat (recorded in 200‐m transect sections, see Newson *et al*. 2009) were covariates. Log detectability was used as an offset. UKBMS records are collected within a 5‐m belt transect so variation in detectability is considerably lower than variation in true abundances (Isaac *et al*. 2011). The model structure for a given species was as follows:

(eqn 1) $$\begin{array}{cc} \text{log}(N_{it}) & =\alpha +\beta_{1}{\text{X1}}_{i}+\beta_{2}{\text{X2}}_{i}\ldots \beta_{n}{\text{Xn}}_{i}+\beta_{t}{\text{Year}}_{t}+\left[\text{log}\left(P_{iv}\right)\right]\\ & \;+{\text{Observation}}_{it}+{\text{Site}}_{i}+50\;\text{km}\;{\text{region}}_{j}+\varepsilon \end{array}$$

where *N*_*it*_ is abundance in site_*i*_ at time_*t*_ in 50‐km region_*j*_, with X1 to Xn being environmental covariates, *P*_*iv*_ is the estimated detection probability at site_*i*_ on visit_*v*_ (birds only) and ε is residual error.

We varied the combinations of environmental variables used in models to evaluate the change in explanatory power when linear features were included (see Table 2 for sets of environmental covariates). Second‐order polynomial terms for all environmental covariates were included to allow for nonlinear relationships with abundance. We refer to environmental variables that were not linear features as ‘land‐cover’ variables.

**Table 2:** Marginal and conditional *R* ^2^ of models of bird and butterfly abundance

Taxa	Explanatory variables	Model structure	Marginal *R* ^2^ (mean ± SE)	Conditional *R* ^2^ (mean ± SE)
Birds	Full	Land cover	0·339 ± 0·068	0·683 ± 0·045
		Land cover + Linear features	0·344 ± 0·066	0·680 ± 0·046
		Land cover * Linear features	0·351 ± 0·066	0·681 ± 0·045
	Agriculture	Land cover	0·168 ± 0·0272	0·626 ± 0·037
		Land cover + Linear features	0·198 ± 0·028	0·612 ± 0·039
		Land cover * Linear features	0·186 ± 0·029	0·609 ± 0·037
	Linear features only		0·146 ± 0·021	0·635 ± 0·048
	Year only		0·129 ± 0·019	0·649 ± 0·048
Butterfly	Full	Land cover	0·206 ± 0·025	0·808 ± 0·022
		Land cover + Linear features	0·219 ± 0·032	0·812 ± 0·022
		Land cover * Linear features	0·221 ± 0·030	0·811 ± 0·022
	Agriculture	Land cover	0·111 ± 0·013	0·797 ± 0·022
		Land cover + Linear features	0·122 ± 0·014	0·796 ± 0·022
		Land cover * Linear features	0·126 ± 0·0135	0·795 ± 0·022
	Linear features only		0·112 ± 0·010	0·810 ± 0·021
	Year only		0·111 ± 0·010	0·812 ± 0·021

+denotes linear features length being included in the model in an additive fashion. *denotes linear features being included as an interaction. Note that *R* ^2^ in mixed effects models do not necessarily increase with additional explanatory variables.

We first assessed whether including total linear features length improved model performance. Models were constructed using all land‐cover variables (the full model set, Table 1), or with only land‐cover variables relating to the extent of agricultural features (the agriculture model set, Table 1). Linear features length was either added to models as a main effect (the additive model), or with interactions with the proportion of arable/horticultural and improved grassland (the interaction model). This interaction term allowed relationships between abundance and woody linear features length to vary in agricultural areas, as linear features may be expected to be more important due to associations of birds and butterflies with farmland hedgerows (e.g. Parish, Lakhani & Sparks 1995).

We assessed whether the addition of linear features increased model explanatory power by calculating *R* ^2^ following Nakagawa & Schielzeth (2013). Changes in *R* ^2^ do not indicate whether improvements in explanatory power justify increases in model complexity. Therefore, we also used AIC to examine whether the more complicated linear features model was more parsimonious than the simpler model. Following Burnham & Anderson (2002), we calculated the AIC weight of each model, which gives a measure of support for a given model being the best of the set of fitted models. From this, we calculated the 95% confidence set of models (the set of best models needed for the cumulative sum of model AIC weights to be 0·95), and the selection probability of variables, defined as the sum of AIC weights of models in which the variable appears (Burnham & Anderson 2002). It should be noted that while the selection probability of main effects can be compared within model sets (e.g. within comparison using all land‐cover variables) as they appear in the same number of models, the selection probability of interaction terms will be smaller because they appear in fewer models, so should not be compared with other variables within model sets. Interaction term selection probabilities can be compared between model sets, as they appear in the same number of models in each set. We also tested whether models including linear features data performed better when tested on independent test data. To do this we split data into independent training (75% of data) and testing (25%) sets, calibrated models to the training set and then tested them on the testing set. This was repeated 100 times. For each model and iteration, we calculated the root‐mean‐squared error on the scale of the linear predictor. This was then used as a response variable in ANOVAs with model set as the explanatory variable, in order to test whether differences in prediction error were significantly different between model sets given the variation in prediction error between iterations. Prediction errors could not be assessed for two bird and one butterfly species due to insufficient data to perform cross‐validation.

Finally, we tested whether estimated woody linear features length was a better descriptor of the environment than total linear features length by selecting for each species the best performing model with a linear features term (i.e. the model with the lowest AIC), then replacing this linear features term with woody linear features length. If the woody liner‐features variable does not improve models, this could be due to species being associated with non‐woody boundary features and/or due to classification errors of woody linear features. We calculated the difference in AIC between these two models to assess whether model performance was improved by including woody linear features.

## Results

Abundance models with land‐cover explanatory variables had moderate explanatory power (mean marginal *R* ^2^ across species in each group: birds = 0·339 ± 0·068 SE, butterflies = 0·206 ± 0·025 SE), although the year term explained a considerable proportion of this variation (marginal *R* ^2^ of models with only year as a fixed effect, birds = 0·129 ± 0·019 SE, butterflies = 0·111 ± 0·010 SE). Including linear feature length in models led to a small increase in explanatory power as measured by marginal *R* ^2^ (mean increase in explanatory power = 4·5%, maximum increase in explanatory power = 29·4%, Tables 2 and S2).

**Figure 1:**
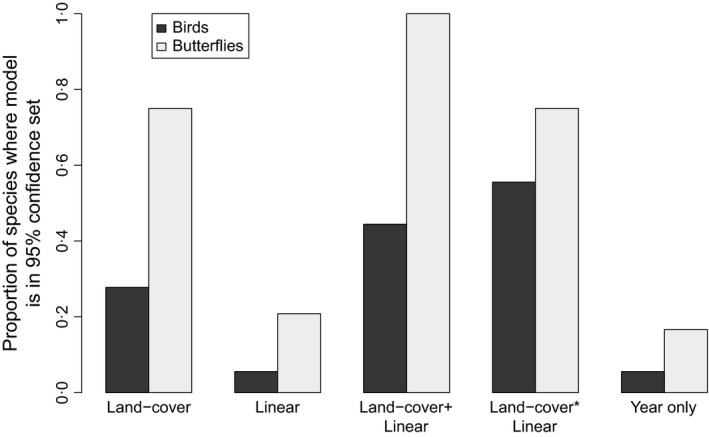
Proportion of species for which models with different variable sets (see Table 1 for terms in each set) were in the 95% confidence set of best supported models. Multiple models for each species can appear in the 95% confidence set.

For 72·2% of bird species (13 of 18 species), the model with the lowest AIC value included linear features length; the same was true for 54·2% (13 out of 24) butterfly species (Table 3). As an additive term, linear features had a selection probability of >0·95 for 12 of the 18 bird species studied, compared with five of 24 butterfly species (Table S2). This indicates that, for birds at least, the increase in explanatory power given by linear features data justified the increase in model complexity. In general, uncertainty over the best model was higher for butterflies than for birds, indicated by the retention of more models in the 95% confidence set (Fig. 1).

**Table 3:** Effect of including linear features as an additive or interaction term on abundance models. Note that prediction errors could not be assessed for two bird species and one butterfly species due to insufficient data to perform cross‐validation

	Number of species	Number of species for which linear features are in the 95% confidence set		Number of species where best model contained linear features	Number of species where linear features term reduced prediction error
Birds	18	Either additive or interaction	14	13	14
		Additive	9	7	14
		Interaction	10	6	12
Butterflies	24	Either additive or interaction	24	13	17
		Additive	24	11	15
		Interaction	18	2	15

**Figure 2:**
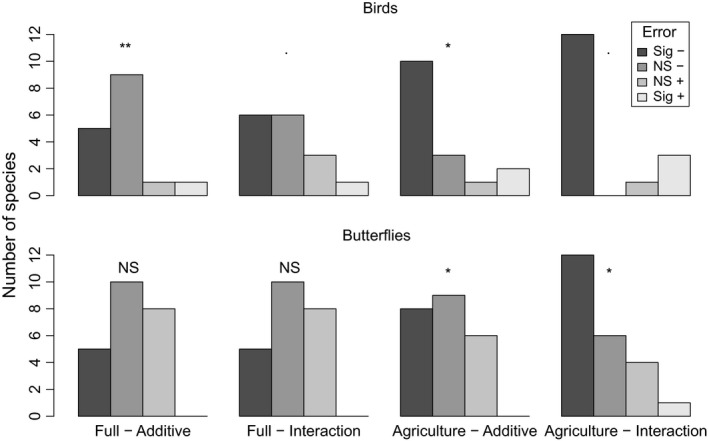
Change in cross‐validation prediction error when linear features length was included in models. Models were fitted using all land‐cover classes (Full) or only those relating to agriculture. Bars show the number of species where prediction error was significantly reduced (Sig −), non‐significantly reduced (NS −), non‐significantly increased (NS +) or significantly increased (Sig +) when linear features length was included. The results of binomial tests, which test whether the proportion of species where linear features improved predictive performance differed from 0·5, are shown above bars. **P < 0·01, *P < 0·05, ˙*P *< 0·1, NS *P *> 0·1).

Inclusion of linear features length reduced cross‐validation prediction error for the majority of bird and butterfly species, with the improvement in model performance most pronounced in birds (Fig. 2, Table 3). However, reductions in prediction error were small (median reduction in prediction error across all species when linear features length was included as an additive term = 0·57%). This was partly due to variation in the importance of linear features and improvements in model predictive performance between species (Table S2), with the change in prediction error with the addition of a linear features additive term varying between a 6·52% reduction (*Sylvia curruca* lesser whitethroat) and a 9·97% increase (*Thymelicus lineola* Essex skipper).

The form of the relationship between abundance and linear features length varied between species (Fig. S1). Just under half of our study species (10 of 18 birds and 10 of 24 butterflies) had positive humped relationships, indicating a preference for intermediate amounts of linear features. Other species (28·6%, e.g. *Columba oenas* stock dove) showed a negative relationship, indicating a preference for areas with few linear features.

Interaction terms between linear features and extent of arable or intensive grassland had selection probabilities >0·95 for five bird species (*Sylvia communis* common whitethroat, *Motacilla flava* yellow wagtail, *Fringilla coelebs* chaffinch, *Carduelis cannabina* linnet and *Emberiza citrinella* yellowhammer), but no butterfly species (Table S2). In all cases, these interactions took the form of increased magnitude of the relationship between abundance and linear features length in agricultural habitats, as well as small shifts in the optimum amount of linear features (Fig. S1). This increase in the magnitude of the relationship with linear features length in agricultural habitats was seen for 10 bird species and 12 butterfly species (Fig. S1), indicating that linear features were often more important determinants of abundance in agricultural landscapes than in other landscapes.

Using woody linear features length instead of total linear features length improved model performance (as judged by lower AIC) for 24 (57%) of the study species, with substantial improvements (∆AIC >4) for 33·3% of species (Table 4). Abundance models of butterflies were more likely to be improved by using woody linear features length (Table 4), although the proportion of species for which models with woody linear features performed better than models with linear features did not differ significantly from 0·5 for both birds and butterflies (binomial tests, *P* = 0·815 and *P* = 0·152, respectively).

**Table 4:** Effect of linear features variable type (all linear features or woody linear features only) on model performance. Differences in the performance of models with different linear features was measured with ΔAIC, with larger values indicating greater support for one model over the other. See Table S4 for results for individual species

	Number of species for which woody linear features term best predicts abundance		Number of species for which all linear features term best predicts abundance	
	ΔAIC ≥4	ΔAIC <4	ΔAIC <4	ΔAIC ≥4
Birds	7	1	1	9
Butterflies	7	9	4	4

## Discussion

This study considered the ability of biotope area and two estimates of linear features to explain butterfly and bird abundance. Inclusion of data on linear features length has the potential to improve species abundance models, as such habitats are known to be important for many species. We found that although increases in model explanatory power were small (mean increase in marginal *R* ^2^ with linear features term = 4·5%; maximum = 29·4%), their inclusion was justified by decreases in model AIC and reduction in cross‐validation prediction errors for most species. Under cross‐validation tests on spatially distinct data, 88% of bird and 67% of butterfly species tested showed reductions in prediction errors when linear features were included in models (Table 1). Abundance models of over half the study species, and particularly butterflies, were further improved by replacing total linear features length with predicted woody linear features length. This demonstrates that even a simple model of linear features length can improve our ability to map biodiversity, and that more sophisticated models that predict whether linear features are hedgerows may lead to further improvements.

Generally, our statistical models had relatively low explanatory power, explaining up to 35% and 22% of the variation in bird and butterfly abundance, respectively (Table S3). There are a number of possible reasons for this: sampling error in species abundance counts, error in the quantification of environmental data and other factors affecting abundance which were not included in models. For example, fine‐scale variation in habitat structure (e.g. species composition) were not included in our models, yet are known to be important for birds and butterflies (e.g. Dennis 2010). Although we recognise the importance of these variables, our approach is pragmatic in testing data that can be gathered relatively cheaply at large spatial scales, but that is still coarse relative to detailed habitat surveys.

Although the addition of linear and woody linear features data improved model performance, the improvements were, perhaps, not as dramatic as may be expected given the evidence from field studies documenting associations between many species and hedgerows (Parish, Lakhani & Sparks 1995; Dover & Sparks 2000). For total linear features length, this may be because the linear features model does not distinguish between different boundary features. Even the woody linear features term is a broad category containing a variety of different habitat structures that differ in their suitability for different species. For example, some species will be positively associated with farmland hedges rather than linear shelterbelts, but the current dataset does not distinguish between type or quality of woody linear feature. Previous studies have found that fine‐scale variation in the structure of hedgerows influence their biodiversity value (Hinsley & Bellamy 2000; Merckx & Berwaerts 2010), with parallel hedges in green lanes having significantly higher butterfly abundances than single hedges (Dover *et al*. 2000), and the inclusion of field‐collected estimates of boundary type and quality has been found to improve models of farmland bird abundance (Siriwardena, Cooke & Sutherland 2012). We did not have data on the quality of hedges or surrounding farmland, so could not capture this fine‐scale variation, but our approach does enable boundary features to be incorporated into landscape scale abundance models. Future developments in LiDAR technology may allow the quantification of hedgerow structure by providing higher resolution data than the 5‐m resolution data used here, giving greater insights into hedgerow quality.

Due to the importance of hedgerows for many species, we expected that including the length of woody linear features would lead to greater improvements to abundance models than including the length of all linear boundary features. However, for 43% of study species, the model including all linear boundary features was better supported than the model with woody linear features extent. This could reflect that non‐woody boundary features will provide important resources for some species (Siriwardena, Cooke & Sutherland 2012). For example, ditches are an important foraging habitat for yellow wagtails (Gilroy *et al*. 2009), and for them, the model containing the length of all linear boundary features performed better than the model with just woody linear features extent. The poorer performance of models with woody linear features length for some species may also result from classification errors, with woody linear features being predicted to occur in places where they were absent, and vice versa. Such errors will impact the performance of abundance models by violating the regression assumption that explanatory variables have been measured without error and could be especially severe if the errors were non‐random (Barry & Elith 2006). Uncertainty over the classification of explanatory variables can be incorporated into models (McInerny & Purves 2011), but requires provision of estimates of uncertainty in GIS datasets. The woody linear features model used currently does not give estimates of uncertainty, but the feasibility of such measures should be considered when probabilistic GIS datasets are created.

Some of our study species would be expected to show strong responses to linear features as previous work has documented their importance. For example, the extent and quality of farmland hedges are known to influence yellowhammer and chaffinch abundance (Bradbury *et al*. 2000; Whittingham *et al*. 2009). Both these species showed strong responses to linear features in this study, but the inclusion of woody linear features length rather than all boundary features improved abundance models for chaffinch but not yellowhammer. The inclusion of linear features also improved abundance models for species such as lapwing *Vanellus vanellus* and stock dove that primarily use resources in field interiors (Murton, Westwood & Isaacson 1964; Vickery, Carter & Fuller 2002); the negative relationship with linear features length for both species is consistent with this. As with birds, butterfly associations sometimes matched and sometimes contrasted with expectations based on previous studies. The abundance of gatekeepers and small heaths in field margins are both positively influenced by hedgerows (Sparks & Parish 1995), but while linear features data improved model predictions for both species, the selection probability of linear features was considerably stronger for small heaths. For some grassland associated species (e.g. meadow brown), the total linear features length term was better supported than woody‐linear features length, which is consistent with these species using grassy boundary features such as banks, but for other species (e.g. brown argus), the woody linear features term was better supported. This could reflect grassland species using hedgerows as movement corridors, and hedges also provide varied microclimates and nectar resources (Dover, Sparks & Greatorex‐Davies 1997; Dennis 2010).

In general, bird abundance models showed greater improvements than butterfly abundance models when linear features data were added. However, the inclusion of woody linear features instead of the total linear features length led to greater improvements for butterflies. This is likely to be due to ecological differences, with many birds deriving benefits from the resources provided by linear features in the wider landscape around sites (Whittingham *et al*. 2009), while butterflies might benefit from hedgerow resources and shelter at a much more local level (Dover, Sparks & Greatorex‐Davies 1997).

Interactions between linear features and agricultural land‐cover were important (i.e. selection probability >0·95) for five bird species, indicating that the relationship between linear features and abundance varied with landscape context. The general form of interactions across species was to increase the importance of linear features in agricultural areas. The particular importance of linear features in agricultural land classes for some species could also be because linear features serve a greater function for connectivity and as habitat in their own right when they cross a hostile agricultural matrix (Davies & Pullin 2007). This supports the intermediate landscape complexity hypothesis (Tscharntke *et al*. 2012), which predicts that interventions to improve landscape quality (e.g. planting hedgerows) are likely to have a greater impact in lower quality landscapes (such as farmland). By showing that, for some species at least, landscape context is important for influencing the importance of linear features, our results hint at the potential to use the linear features dataset to identify areas where linear features are particularly important.

In this study we focus on birds and butterflies because large monitoring schemes means that it is possible to assess the national‐scale importance of linear features. However, linear features are important for many other taxa, including invertebrates other than butterflies (Maudsley 2000), so linear features extent could potentially improve abundance models for many taxa. It may also be possible to extend the linear features models used here to determine the identity of non‐woody boundary features. For example, improved knowledge of the distribution of ditches could help model the distribution of wetland‐associated biodiversity in agricultural landscapes (Mossman, Panter & Dolman 2015). Modelling approaches using linear features data could be extended to quantify connectivity between habitats. For example, if linear features are assumed to be corridors allowing movement through matrix habitat, then they can be used in circuit theory models to quantify connectivity among habitat patches (McRae *et al*. 2008). Such models can then be combined with movement data to test the value of linear features for enhancing connectivity for different taxa. While such tests can be performed at small spatial scales using field‐collected linear features data, or at large scales by quantifying fragmentation of woodland land cover (e.g. Newson *et al*. 2014), the availability of national‐scale data potentially allows connectivity networks to be mapped at broad spatial scales, facilitating tests of their utility for delivering biodiversity benefits as well as design of evidence‐based connectivity networks. Finally, we note that although the linear features models used in this study are based on UK mapping and remote‐sensing data, similar models could be developed in other countries providing there is a spatial framework that can be used to identify field boundaries, and an estimate of canopy height which can be used to predict whether linear boundary features are woody. This may be particularly valuable in areas where woody linear features are proposed as corridors to mitigate against forest fragmentation (e.g. Lees & Peres 2008).

In conclusion, linear features such as hedgerows are known to be important for many taxa, so large‐scale GIS data on their distribution and extent would be expected to improve models of the abundance of birds and butterflies. Our results confirm this hypothesis, although the extent of improvements varied between species. Linear features data can be used in a variety of modelling applications, for example, examining the extent to which the importance of linear features varies spatially and between taxa. This could assist targeting of agri‐environment schemes and other hedgerow planting incentives, in order to provide linear features where they are most needed. Further developments to improve the classification accuracy of the GIS dataset are likely to further improve its utility for end‐users.

## Authors' contributions

T.O. conceived the study; M.S., J.P.H., S.N. and T.O. designed the study methodology; P.S. developed the linear features model; T.B., J.P.H. and S.N. coordinated data collection; M.S. analysed the data with input from all other authors; M.S., J.P.H., S.N. and T.O. led the writing of the manuscript with contributions from all authors.

## Data accessibility

National‐scale linear features data are deposited at https://doi.org/10.5285/d7da6cb9-104b-4dbc-b709-c1f7ba94fb16 (Scholefield *et al*. 2016b). Bird and butterfly abundance data are deposited at https://doi.org/10.5061/dryad.m5g04 (Sullivan *et al*. 2017).

## Supporting information


**Fig. S1.** Relationship between species abundance and woody linear features length for (a) birds and (b) butterflies.

**Table S1.** Environmental characteristics in 1‐km radius buffers around UK butterfly monitoring scheme (UKBMS) and breeding bird survey (BBS) transects.

**Table S2.** Species investigated in this study, and importance of woody linear features at explaining and predicting their abundance.

**Table S3.** *R* ^2^ and AIC of individual models for each species.

**Table S4.** Effect of linear features variable type on abundance models for each species.
