# Supplementary material for: A national‐scale model of linear features improves predictions of farmland biodiversity
Source: J Appl Ecol. 2017 May 7;54(6):1776–84. doi: 10.1111/1365-2664.12912 (PMC5697618; doi:10.1111/1365-2664.12912)
Supplement: Supplementary file 1 — Fig. S1. Relationship between species abundance and woody linear features length for (a) birds and (b) butterflies. [file JPE-54-1776-s001.docx]

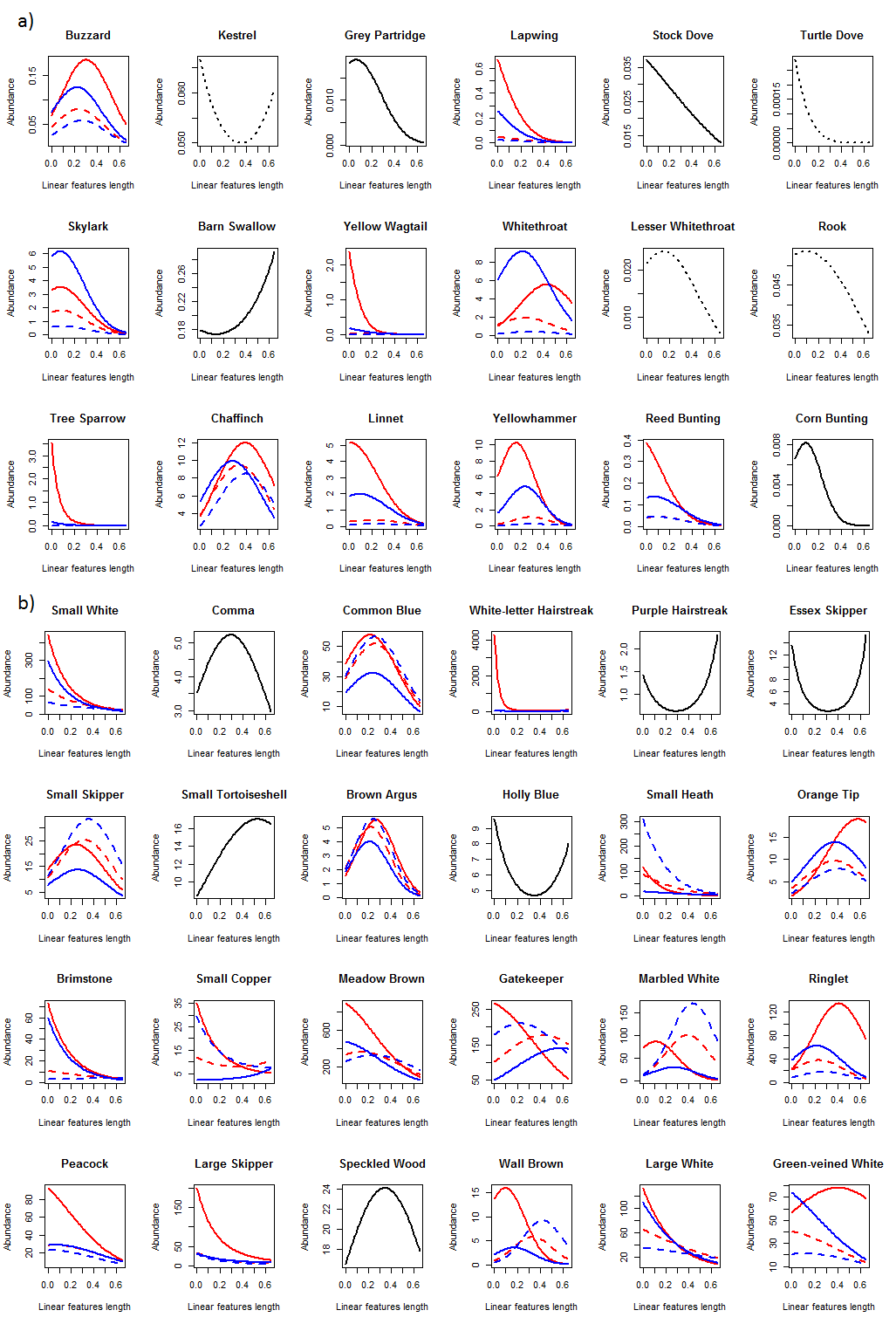


**Figure S1.** Relationship between species abundance and woody linear features length for (a) birds and (b) butterflies. Response curves are shown from Poisson generalized linear mixed effects models containing all explanatory variables and interactions (see Table 1). Where interaction terms were present in the 95% confidence set of best supported models, response curves show modelled relationships with linear features length when arable (blue) or improved grassland (red) was varied between covering 20% (dashed lines) and 80% (solid lines) of the 1km radius around monitoring sites. Other variables were held at their mean values. Where linear features terms were only present in the 95 % confidence set as a main effect, response curves show relationships with linear features whilst holding all other variables at their mean (solid black line). Response curves are plotted with black dotted lines where no linear features terms were present in the 95 % confidence set.
