# Supplementary material for: A national‐scale model of linear features improves predictions of farmland biodiversity
Source: J Appl Ecol. 2017 May 7;54(6):1776–84. doi: 10.1111/1365-2664.12912 (PMC5697618; doi:10.1111/1365-2664.12912)
Supplement: Supplementary file 2 — Table S1. Environmental characteristics in 1‐km radius buffers around UK butterfly monitoring scheme (UKBMS) and breeding bird survey (BBS) transects. [file JPE-54-1776-s002.docx]

**Table S1.** Environmental characteristics in 1km radius buffers around UK butterfly monitoring scheme (UKBMS) and breeding bird survey (BBS) transects.

| Environmental variable | UKBMS (mean ± SD) |  | BBS (mean ± SD) |  |
| --- | --- | --- | --- | --- |
| Arable (proportion) | 0.224 | ± 0.208 | 0.331 | ± 0.289 |
| Improved grassland (proportion) | 0.273 | ± 0.177 | 0.273 | ± 0.203 |
| Rough grassland (proportion) | 0.057 | ± 0.073 | 0.045 | ± 0.068 |
| Natural grassland (proportion) | 0.028 | ± 0.073 | 0.047 | ± 0.074 |
| Calcareous grassland (proportion) | 0.004 | ± 0.026 | 0.008 | ± 0.025 |
| Fen, marsh and swamp (proportion) | 0.003 | ± 0.022 | 0.034 | ± 0.113 |
| Heath and bog proportion) | 0.033 | ± 0.098 | 0.038 | ± 0.120 |
| Urban and suburban (proportion) | 0.109 | ± 0.187 | 0.026 | ± 0.082 |
| Freshwater (proportion) | 0.012 | ± 0.044 | 0.002 | ± 0.018 |
| Broadleaved woodland (proportion) | 0.168 | ± 0.153 | 0.003 | ± 0.029 |
| Coniferous woodland (proportion) | 0.042 | ± 0.098 | 0.076 | ± 0.095 |
| Altitude (m) | 103.9 | ± 87.3 | 116.5 | ± 113.0 |
| Linear features length (m/100000) | 0.406 | ± 0.103 | 0.374 | ± 0.117 |
| Woody linear features length (m/100000) | 0.085 | ± 0.054 | 0.083 | ± 0.056 |
