# Supplementary material for: A national‐scale model of linear features improves predictions of farmland biodiversity
Source: J Appl Ecol. 2017 May 7;54(6):1776–84. doi: 10.1111/1365-2664.12912 (PMC5697618; doi:10.1111/1365-2664.12912)
Supplement: Supplementary file 3 — Table S2. Species investigated in this study, and importance of woody linear features at explaining and predicting their abundance. [file JPE-54-1776-s003.docx]

**Table S2.** Species investigated in this study, and importance of woody linear features at explaining and predicting their abundance. Species with NA for cross-validation error were recorded in too few locations to split data into training and testing sets and have sufficient data to fit the models. MSE is mean squared error when models were validated on independent testing data.

| Common name | Scientific name | Selection probability of woody linear features | | Cross validation error (√MSE) | | |  |
| --- | --- | --- | --- | --- | --- | --- | --- |
|  |  | Additive term | Interaction term | Full landscape model | Δ with additive linear features (%) | Δ with linear features interaction (%) | |
| Birds |  |  |  |  |  |  | |
| Grey Partridge | *Perdix perdix* | 1.00 | 0.00 | 5.34 | -3.77 | -5.71 | |
| Yellowhammer | *Emberiza citrinella* | 1.00 | 1.00 | 1.48 | -1.28 | -2.41 | |
| Skylark | *Alauda arvensis* | 1.00 | 0.13 | 1.57 | -0.04 | -0.40 | |
| Corn Bunting | *Miliaria calandra* | 1.00 | 0.00 | NA | NA | NA | |
| Chaffinch | *Fringilla coelebs* | 1.00 | 1.00 | 0.75 | -1.25 | -1.53 | |
| Reed Bunting | *Emberiza schoeniclus* | 1.00 | 0.30 | 3.36 | -0.60 | -1.31 | |
| Lapwing | *Vanellus vanellus* | 1.00 | 0.94 | 5.21 | -0.63 | -0.74 | |
| Yellow Wagtail | *Motacilla flava* | 1.00 | 1.00 | 12.47 | 8.07 | 9.24 | |
| Buzzard | *Buteo buteo* | 1.00 | 0.27 | 2.73 | -1.66 | -0.82 | |
| Linnet | *Carduelis cannabina* | 1.00 | 1.00 | 2.38 | -0.42 | -0.58 | |
| Common Whitethroat | *Sylvia communis* | 1.00 | 0.98 | 2.50 | 1.41 | 0.88 | |
| Barn Swallow | *Hirundo rustica* | 0.99 | 0.00 | 2.23 | -0.98 | -2.24 | |
| Tree Sparrow | *Passer montanus* | 0.77 | 0.18 | 12.35 | -3.92 | -0.68 | |
| Stock Dove | *Columba oenas* | 0.06 | 0.00 | 3.75 | -0.35 | 0.31 | |
| Lesser Whitethroat | *Sylvia curruca* | 0.00 | 0.00 | 4.66 | -6.52 | -7.61 | |
| Kestrel | *Falco tinnunculus* | 0.00 | 0.00 | 3.20 | -0.28 | 0.21 | |
| Rook | *Corvus frugilegus* | 0.00 | 0.00 | 4.40 | -2.25 | -1.51 | |
| Turtle Dove | *Streptopelia turtur* | 0.00 | 0.00 | NA | NA | NA | |
| Butterflies |  |  |  |  |  |  | |
| Small Heath | *Coenonympha pamphilus* | 0.99 | 0.19 | 2.01 | -1.88 | -2.07 | |
| Peacock | *Inachis io* | 0.99 | 0.22 | 1.17 | -0.41 | -0.26 | |
| Brown Argus | *Aricia agestis* | 0.98 | 0.12 | 1.68 | -0.63 | -0.40 | |
| Large Skipper | *Ochlodes sylvanus* | 0.97 | 0.16 | 1.29 | -1.13 | -0.98 | |
| Small White | *Pieris rapae* | 0.96 | 0.39 | 1.16 | -0.69 | -0.68 | |
| Wall Brown | *Lasiommata megera* | 0.91 | 0.83 | 2.15 | -2.10 | -2.68 | |
| Small Copper | *Lycaena phlaeas* | 0.91 | 0.62 | 1.39 | -0.47 | -0.94 | |
| Essex Skipper | *Thymelicus lineola* | 0.87 | 0.02 | 2.85 | 9.97 | -15.22 | |
| Common Blue | *Polyommatus icarus* | 0.84 | 0.10 | 1.59 | -0.75 | -0.94 | |
| Meadow Brown | *Maniola jurtina* | 0.77 | 0.27 | 1.17 | 0.35 | -0.10 | |
| Large White | *Pieris brassicae* | 0.68 | 0.28 | 1.06 | 0.34 | 0.49 | |
| Ringlet | *Aphantopus hyperantus* | 0.64 | 0.20 | 1.49 | -0.61 | -0.72 | |
| Gatekeeper | *Pyronia tithonus* | 0.63 | 0.26 | 1.18 | -0.57 | -0.94 | |
| White-letter Hairstreak | *Satyrium w-album* | 0.61 | 0.05 | NA | NA | NA | |
| Orange Tip | *Anthocharis cardamines* | 0.47 | 0.12 | 1.08 | -0.22 | 0.12 | |
| Purple Hairstreak | *Neozephrus quercus* | 0.47 | 0.02 | 1.89 | 0.17 | 0.67 | |
| Marbled White | *Melanargia galathea* | 0.36 | 0.16 | 1.82 | -0.05 | -0.48 | |
| Brimstone | *Gonepteryx rhamni* | 0.27 | 0.16 | 1.47 | -0.91 | -1.58 | |
| Comma | *Polygonia c-album* | 0.25 | 0.03 | 1.13 | -0.40 | -0.27 | |
| Green-veined White | *Pieris napi* | 0.23 | 0.05 | 1.35 | 0.53 | 0.30 | |
| Small Skipper | *Thymelicus sylvestris* | 0.22 | 0.05 | 1.84 | -0.57 | 0.20 | |
| Small Tortoiseshell | *Aglais urticae* | 0.21 | 0.04 | 1.26 | 0.22 | 0.37 | |
| Holly Blue | *Celastrina argiolus* | 0.18 | 0.02 | 1.08 | 0.77 | 0.37 | |
| Speckled Wood | *Pararge aegeria* | 0.15 | 0.03 | 1.33 | 0.04 | 0.19 | |
