# Supplementary material for: A national‐scale model of linear features improves predictions of farmland biodiversity
Source: J Appl Ecol. 2017 May 7;54(6):1776–84. doi: 10.1111/1365-2664.12912 (PMC5697618; doi:10.1111/1365-2664.12912)
Supplement: Supplementary file 4 — Table S3. R 2 and AIC of individual models for each species. [file JPE-54-1776-s004.docx]

**Table S3.** *R*^2^ and AIC of individual models for each species.

| Species | Explanatory variables | Model structure | Marginal *R*^2^ | Conditional *R*^2^ | AIC |
| --- | --- | --- | --- | --- | --- |
| Buzzard | Full | Land-cover | 0.075 | 0.528 | 10550.0 |
| Buzzard | Full | Land-cover + Linear | 0.081 | 0.531 | 10530.1 |
| Buzzard | Full | Land-cover * Linear | 0.083 | 0.533 | 10532.0 |
| Buzzard | Agriculture | Land-cover | 0.044 | 0.491 | 10590.2 |
| Buzzard | Agriculture | Land-cover + Linear | 0.054 | 0.484 | 10559.5 |
| Buzzard | Agriculture | Land-cover * Linear | 0.055 | 0.498 | 10558.3 |
| Buzzard | Linear |  | 0.055 | 0.487 | 10608.5 |
| Buzzard | Year |  | 0.053 | 0.510 | 10676.8 |
| Corn Bunting | Full | Land-cover | 0.932 | 0.967 | 4233.7 |
| Corn Bunting | Full | Land-cover + Linear | 0.954 | 0.977 | 4198.0 |
| Corn Bunting | Full | Land-cover * Linear | 0.937 | 0.968 | 4199.9 |
| Corn Bunting | Agriculture | Land-cover | 0.182 | 0.669 | 4252.9 |
| Corn Bunting | Agriculture | Land-cover + Linear | 0.259 | 0.707 | 4162.1 |
| Corn Bunting | Agriculture | Land-cover * Linear | 0.273 | 0.666 | 4238.7 |
| Corn Bunting | Linear |  | 0.151 | 0.815 | 4184.8 |
| Corn Bunting | Year |  | 0.175 | 0.791 | 4382.2 |
| Chaffinch | Full | Land-cover | 0.306 | 0.894 | 68268.6 |
| Chaffinch | Full | Land-cover + Linear | 0.317 | 0.893 | 68232.8 |
| Chaffinch | Full | Land-cover * Linear | 0.326 | 0.893 | 68213.2 |
| Chaffinch | Agriculture | Land-cover | 0.204 | 0.894 | 68563.7 |
| Chaffinch | Agriculture | Land-cover + Linear | 0.233 | 0.892 | 68481.8 |
| Chaffinch | Agriculture | Land-cover * Linear | 0.253 | 0.891 | 68427.8 |
| Chaffinch | Linear |  | 0.198 | 0.915 | 68945.5 |
| Chaffinch | Year |  | 0.168 | 0.928 | 69214.7 |
| Kestrel | Full | Land-cover | 0.097 | 0.301 | 8235.6 |
| Kestrel | Full | Land-cover + Linear | 0.093 | 0.286 | 8250.4 |
| Kestrel | Full | Land-cover * Linear | 0.090 | 0.281 | 8248.3 |
| Kestrel | Agriculture | Land-cover | 0.037 | 0.262 | 8169.2 |
| Kestrel | Agriculture | Land-cover + Linear | 0.069 | 0.223 | 8211.9 |
| Kestrel | Agriculture | Land-cover * Linear | 0.058 | 0.270 | 8274.6 |
| Kestrel | Linear |  | 0.042 | 0.231 | 8242.9 |
| Kestrel | Year |  | 0.037 | 0.173 | 8190.9 |
| Lapwing | Full | Land-cover | 0.062 | 0.564 | 14448.7 |
| Lapwing | Full | Land-cover + Linear | 0.080 | 0.565 | 14414.8 |
| Lapwing | Full | Land-cover * Linear | 0.078 | 0.564 | 14409.4 |
| Lapwing | Agriculture | Land-cover | 0.030 | 0.612 | 14551.4 |
| Lapwing | Agriculture | Land-cover + Linear | 0.055 | 0.563 | 14465.6 |
| Lapwing | Agriculture | Land-cover * Linear | 0.051 | 0.581 | 14479.5 |
| Lapwing | Linear |  | 0.050 | 0.581 | 14543.1 |
| Lapwing | Year |  | 0.044 | 0.554 | 14551.9 |
| Linnet | Full | Land-cover | 0.184 | 0.636 | 31936.0 |
| Linnet | Full | Land-cover + Linear | 0.199 | 0.644 | 31930.6 |
| Linnet | Full | Land-cover * Linear | 0.219 | 0.658 | 31918.0 |
| Linnet | Agriculture | Land-cover | 0.167 | 0.628 | 32012.1 |
| Linnet | Agriculture | Land-cover + Linear | 0.189 | 0.636 | 31967.7 |
| Linnet | Agriculture | Land-cover * Linear | 0.172 | 0.637 | 31965.6 |
| Linnet | Linear |  | 0.155 | 0.699 | 32383.9 |
| Linnet | Year |  | 0.161 | 0.682 | 32530.3 |
| Lesser Whitethroat | Full | Land-cover | 0.343 | 0.622 | 7729.6 |
| Lesser Whitethroat | Full | Land-cover + Linear | 0.375 | 0.630 | 7738.1 |
| Lesser Whitethroat | Full | Land-cover * Linear | 0.362 | 0.623 | 7743.7 |
| Lesser Whitethroat | Agriculture | Land-cover | 0.255 | 0.631 | 7698.1 |
| Lesser Whitethroat | Agriculture | Land-cover + Linear | 0.211 | 0.548 | 7752.1 |
| Lesser Whitethroat | Agriculture | Land-cover * Linear | 0.230 | 0.553 | 7760.1 |
| Lesser Whitethroat | Linear |  | 0.198 | 0.608 | 7941.1 |
| Lesser Whitethroat | Year |  | 0.169 | 0.640 | 7961.3 |
| Grey Partridge | Full | Land-cover | 0.134 | 0.447 | 7645.8 |
| Grey Partridge | Full | Land-cover + Linear | 0.154 | 0.447 | 7617.3 |
| Grey Partridge | Full | Land-cover * Linear | 0.173 | 0.457 | 7614.8 |
| Grey Partridge | Agriculture | Land-cover | 0.144 | 0.432 | 7582.8 |
| Grey Partridge | Agriculture | Land-cover + Linear | 0.119 | 0.420 | 7605.3 |
| Grey Partridge | Agriculture | Land-cover * Linear | 0.133 | 0.441 | 7615.8 |
| Grey Partridge | Linear |  | 0.066 | 0.180 | 7356.6 |
| Grey Partridge | Year |  | 0.099 | 0.465 | 7637.8 |
| Reed Bunting | Full | Land-cover | 0.144 | 0.666 | 13632.7 |
| Reed Bunting | Full | Land-cover + Linear | 0.173 | 0.666 | 13583.7 |
| Reed Bunting | Full | Land-cover * Linear | 0.169 | 0.659 | 13585.4 |
| Reed Bunting | Agriculture | Land-cover | 0.094 | 0.659 | 13757.2 |
| Reed Bunting | Agriculture | Land-cover + Linear | 0.137 | 0.671 | 13675.4 |
| Reed Bunting | Agriculture | Land-cover * Linear | 0.131 | 0.660 | 13675.4 |
| Reed Bunting | Linear |  | 0.115 | 0.703 | 13895.5 |
| Reed Bunting | Year |  | 0.108 | 0.724 | 14010.9 |
| Rook | Full | Land-cover | 0.120 | 0.534 | 34194.8 |
| Rook | Full | Land-cover + Linear | 0.097 | 0.523 | 34188.1 |
| Rook | Full | Land-cover * Linear | 0.136 | 0.545 | 34181.8 |
| Rook | Agriculture | Land-cover | 0.127 | 0.589 | 34023.3 |
| Rook | Agriculture | Land-cover + Linear | 0.135 | 0.545 | 34159.2 |
| Rook | Agriculture | Land-cover * Linear | 0.132 | 0.543 | 34186.0 |
| Rook | Linear |  | 0.123 | 0.544 | 34198.7 |
| Rook | Year |  | 0.094 | 0.542 | 34342.1 |
| Skylark | Full | Land-cover | 0.336 | 0.836 | 39933.0 |
| Skylark | Full | Land-cover + Linear | 0.386 | 0.831 | 39764.2 |
| Skylark | Full | Land-cover * Linear | 0.391 | 0.832 | 39767.9 |
| Skylark | Agriculture | Land-cover | 0.242 | 0.835 | 40273.7 |
| Skylark | Agriculture | Land-cover + Linear | 0.359 | 0.832 | 39989.8 |
| Skylark | Agriculture | Land-cover * Linear | 0.350 | 0.827 | 39991.2 |
| Skylark | Linear |  | 0.322 | 0.840 | 40391.1 |
| Skylark | Year |  | 0.264 | 0.870 | 41118.9 |
| Stock Dove | Full | Land-cover | 0.068 | 0.452 | 14750.2 |
| Stock Dove | Full | Land-cover + Linear | 0.078 | 0.458 | 14747.6 |
| Stock Dove | Full | Land-cover * Linear | 0.084 | 0.461 | 14754.0 |
| Stock Dove | Agriculture | Land-cover | 0.061 | 0.444 | 14595.8 |
| Stock Dove | Agriculture | Land-cover + Linear | 0.123 | 0.459 | 14601.3 |
| Stock Dove | Agriculture | Land-cover * Linear | 0.080 | 0.457 | 14781.6 |
| Stock Dove | Linear |  | 0.078 | 0.469 | 14819.2 |
| Stock Dove | Year |  | 0.069 | 0.428 | 14689.4 |
| Swallow | Full | Land-cover | 0.164 | 0.635 | 31310.4 |
| Swallow | Full | Land-cover + Linear | 0.162 | 0.631 | 31301.1 |
| Swallow | Full | Land-cover * Linear | 0.156 | 0.627 | 31303.0 |
| Swallow | Agriculture | Land-cover | 0.124 | 0.608 | 31287.6 |
| Swallow | Agriculture | Land-cover + Linear | 0.157 | 0.620 | 31278.1 |
| Swallow | Agriculture | Land-cover * Linear | 0.155 | 0.622 | 31294.9 |
| Swallow | Linear |  | 0.147 | 0.644 | 31657.5 |
| Swallow | Year |  | 0.140 | 0.660 | 31780.7 |
| Turtle Dove | Full | Land-cover | 0.779 | 0.897 | 3532.9 |
| Turtle Dove | Full | Land-cover + Linear | 0.751 | 0.919 | 3625.1 |
| Turtle Dove | Full | Land-cover * Linear | 0.798 | 0.907 | 3530.6 |
| Turtle Dove | Agriculture | Land-cover | 0.068 | 0.679 | 3623.2 |
| Turtle Dove | Agriculture | Land-cover + Linear | 0.116 | 0.554 | 3438.8 |
| Turtle Dove | Agriculture | Land-cover * Linear | 0.045 | 0.549 | 3486.7 |
| Turtle Dove | Linear |  | 0.037 | 0.631 | 3501.0 |
| Turtle Dove | Year |  | 0.022 | 0.424 | 3191.2 |
| Tree Sparrow | Full | Land-cover | 0.859 | 0.949 | 6793.1 |
| Tree Sparrow | Full | Land-cover + Linear | 0.865 | 0.950 | 6787.0 |
| Tree Sparrow | Full | Land-cover * Linear | 0.877 | 0.954 | 6790.5 |
| Tree Sparrow | Agriculture | Land-cover | 0.183 | 0.721 | 6772.1 |
| Tree Sparrow | Agriculture | Land-cover + Linear | 0.204 | 0.717 | 6770.3 |
| Tree Sparrow | Agriculture | Land-cover * Linear | 0.195 | 0.710 | 6772.7 |
| Tree Sparrow | Linear |  | 0.185 | 0.722 | 6952.4 |
| Tree Sparrow | Year |  | 0.100 | 0.850 | 6918.1 |
| Whitethroat | Full | Land-cover | 0.373 | 0.768 | 65363.9 |
| Whitethroat | Full | Land-cover + Linear | 0.380 | 0.773 | 65356.0 |
| Whitethroat | Full | Land-cover * Linear | 0.392 | 0.774 | 65348.3 |
| Whitethroat | Agriculture | Land-cover | 0.354 | 0.769 | 65401.2 |
| Whitethroat | Agriculture | Land-cover + Linear | 0.376 | 0.768 | 65398.3 |
| Whitethroat | Agriculture | Land-cover * Linear | 0.373 | 0.771 | 65392.8 |
| Whitethroat | Linear |  | 0.263 | 0.842 | 66338.0 |
| Whitethroat | Year |  | 0.245 | 0.852 | 66474.8 |
| Yellowhammer | Full | Land-cover | 0.473 | 0.814 | 30433.1 |
| Yellowhammer | Full | Land-cover + Linear | 0.501 | 0.816 | 30359.0 |
| Yellowhammer | Full | Land-cover * Linear | 0.511 | 0.816 | 30345.8 |
| Yellowhammer | Agriculture | Land-cover | 0.462 | 0.813 | 30473.8 |
| Yellowhammer | Agriculture | Land-cover + Linear | 0.499 | 0.819 | 30387.1 |
| Yellowhammer | Agriculture | Land-cover * Linear | 0.490 | 0.811 | 30382.2 |
| Yellowhammer | Linear |  | 0.333 | 0.871 | 31467.7 |
| Yellowhammer | Year |  | 0.296 | 0.886 | 31947.7 |
| Yellow Wagtail | Full | Land-cover | 0.660 | 0.784 | 4078.0 |
| Yellow Wagtail | Full | Land-cover + Linear | 0.541 | 0.698 | 4045.0 |
| Yellow Wagtail | Full | Land-cover * Linear | 0.542 | 0.698 | 4046.4 |
| Yellow Wagtail | Agriculture | Land-cover | 0.253 | 0.537 | 4018.4 |
| Yellow Wagtail | Agriculture | Land-cover + Linear | 0.272 | 0.550 | 4014.0 |
| Yellow Wagtail | Agriculture | Land-cover * Linear | 0.179 | 0.466 | 3983.9 |
| Yellow Wagtail | Linear |  | 0.103 | 0.641 | 4027.6 |
| Yellow Wagtail | Year |  | 0.084 | 0.697 | 4065.3 |
| Small White | Full | Land-cover | 0.298 | 0.774 | 16815.8 |
| Small White | Full | Land-cover + Linear | 0.309 | 0.775 | 16811.2 |
| Small White | Full | Land-cover * Linear | 0.311 | 0.774 | 16811.5 |
| Small White | Agriculture | Land-cover | 0.262 | 0.775 | 16825.1 |
| Small White | Agriculture | Land-cover + Linear | 0.280 | 0.776 | 16812.8 |
| Small White | Agriculture | Land-cover * Linear | 0.281 | 0.775 | 16815.8 |
| Small White | Linear |  | 0.230 | 0.815 | 16930.5 |
| Small White | Year |  | 0.220 | 0.823 | 16960.4 |
| Comma | Full | Land-cover | 0.225 | 0.777 | 11468.7 |
| Comma | Full | Land-cover + Linear | 0.227 | 0.775 | 11471.2 |
| Comma | Full | Land-cover * Linear | 0.226 | 0.776 | 11475.2 |
| Comma | Agriculture | Land-cover | 0.163 | 0.773 | 11491.9 |
| Comma | Agriculture | Land-cover + Linear | 0.165 | 0.773 | 11493.2 |
| Comma | Agriculture | Land-cover * Linear | 0.170 | 0.773 | 11492.6 |
| Comma | Linear |  | 0.144 | 0.808 | 11548.2 |
| Comma | Year |  | 0.145 | 0.807 | 11545.5 |
| Common Blue | Full | Land-cover | 0.263 | 0.875 | 16519.6 |
| Common Blue | Full | Land-cover + Linear | 0.271 | 0.873 | 16516.6 |
| Common Blue | Full | Land-cover * Linear | 0.271 | 0.873 | 16520.6 |
| Common Blue | Agriculture | Land-cover | 0.156 | 0.875 | 16582.7 |
| Common Blue | Agriculture | Land-cover + Linear | 0.176 | 0.875 | 16571.6 |
| Common Blue | Agriculture | Land-cover * Linear | 0.176 | 0.875 | 16574.9 |
| Common Blue | Linear |  | 0.163 | 0.885 | 16622.6 |
| Common Blue | Year |  | 0.162 | 0.886 | 16634.8 |
| White-letter Hairstreak | Full | Land-cover | 0.702 | 0.830 | 626.7 |
| White-letter Hairstreak | Full | Land-cover + Linear | 0.882 | 0.928 | 621.5 |
| White-letter Hairstreak | Full | Land-cover * Linear | 0.854 | 0.910 | 623.4 |
| White-letter Hairstreak | Agriculture | Land-cover | 0.145 | 0.542 | 605.8 |
| White-letter Hairstreak | Agriculture | Land-cover + Linear | 0.182 | 0.536 | 602.4 |
| White-letter Hairstreak | Agriculture | Land-cover * Linear | 0.186 | 0.526 | 603.2 |
| White-letter Hairstreak | Linear |  | 0.164 | 0.563 | 598.6 |
| White-letter Hairstreak | Year |  | 0.155 | 0.588 | 599.1 |
| Purple Hairstreak | Full | Land-cover | 0.130 | 0.672 | 2706.5 |
| Purple Hairstreak | Full | Land-cover + Linear | 0.140 | 0.671 | 2706.8 |
| Purple Hairstreak | Full | Land-cover * Linear | 0.138 | 0.667 | 2709.7 |
| Purple Hairstreak | Agriculture | Land-cover | 0.068 | 0.669 | 2694.3 |
| Purple Hairstreak | Agriculture | Land-cover + Linear | 0.077 | 0.673 | 2694.4 |
| Purple Hairstreak | Agriculture | Land-cover * Linear | 0.073 | 0.669 | 2697.9 |
| Purple Hairstreak | Linear |  | 0.069 | 0.686 | 2692.9 |
| Purple Hairstreak | Year |  | 0.070 | 0.682 | 2692.4 |
| Essex Skipper | Full | Land-cover | 0.152 | 0.715 | 2777.9 |
| Essex Skipper | Full | Land-cover + Linear | 0.164 | 0.713 | 2778.2 |
| Essex Skipper | Full | Land-cover * Linear | 0.165 | 0.711 | 2781.3 |
| Essex Skipper | Agriculture | Land-cover | 0.082 | 0.706 | 2770.3 |
| Essex Skipper | Agriculture | Land-cover + Linear | 0.097 | 0.698 | 2767.0 |
| Essex Skipper | Agriculture | Land-cover * Linear | 0.106 | 0.693 | 2767.6 |
| Essex Skipper | Linear |  | 0.099 | 0.708 | 2760.5 |
| Essex Skipper | Year |  | 0.094 | 0.726 | 2764.4 |
| Small Skipper | Full | Land-cover | 0.123 | 0.714 | 10298.3 |
| Small Skipper | Full | Land-cover + Linear | 0.125 | 0.714 | 10301.4 |
| Small Skipper | Full | Land-cover * Linear | 0.128 | 0.714 | 10303.8 |
| Small Skipper | Agriculture | Land-cover | 0.066 | 0.720 | 10303.0 |
| Small Skipper | Agriculture | Land-cover + Linear | 0.069 | 0.720 | 10305.6 |
| Small Skipper | Agriculture | Land-cover * Linear | 0.071 | 0.719 | 10308.8 |
| Small Skipper | Linear |  | 0.067 | 0.734 | 10309.7 |
| Small Skipper | Year |  | 0.068 | 0.730 | 10307.0 |
| Small Tortoiseshell | Full | Land-cover | 0.190 | 0.720 | 11369.4 |
| Small Tortoiseshell | Full | Land-cover + Linear | 0.189 | 0.719 | 11372.4 |
| Small Tortoiseshell | Full | Land-cover * Linear | 0.189 | 0.719 | 11375.5 |
| Small Tortoiseshell | Agriculture | Land-cover | 0.110 | 0.721 | 11402.2 |
| Small Tortoiseshell | Agriculture | Land-cover + Linear | 0.120 | 0.721 | 11398.6 |
| Small Tortoiseshell | Agriculture | Land-cover * Linear | 0.124 | 0.723 | 11397.2 |
| Small Tortoiseshell | Linear |  | 0.115 | 0.744 | 11436.5 |
| Small Tortoiseshell | Year |  | 0.114 | 0.745 | 11440.4 |
| Brown Argus | Full | Land-cover | 0.178 | 0.825 | 6800.7 |
| Brown Argus | Full | Land-cover + Linear | 0.202 | 0.828 | 6795.4 |
| Brown Argus | Full | Land-cover * Linear | 0.202 | 0.827 | 6799.4 |
| Brown Argus | Agriculture | Land-cover | 0.113 | 0.827 | 6800.4 |
| Brown Argus | Agriculture | Land-cover + Linear | 0.145 | 0.827 | 6791.8 |
| Brown Argus | Agriculture | Land-cover * Linear | 0.140 | 0.826 | 6795.5 |
| Brown Argus | Linear |  | 0.132 | 0.836 | 6798.0 |
| Brown Argus | Year |  | 0.131 | 0.837 | 6805.0 |
| Holly Blue | Full | Land-cover | 0.171 | 0.596 | 6241.6 |
| Holly Blue | Full | Land-cover + Linear | 0.164 | 0.595 | 6245.3 |
| Holly Blue | Full | Land-cover * Linear | 0.163 | 0.595 | 6248.4 |
| Holly Blue | Agriculture | Land-cover | 0.142 | 0.603 | 6229.4 |
| Holly Blue | Agriculture | Land-cover + Linear | 0.140 | 0.598 | 6232.8 |
| Holly Blue | Agriculture | Land-cover * Linear | 0.141 | 0.594 | 6235.4 |
| Holly Blue | Linear |  | 0.138 | 0.606 | 6232.6 |
| Holly Blue | Year |  | 0.138 | 0.604 | 6229.6 |
| Small Heath | Full | Land-cover | 0.241 | 0.934 | 11855.3 |
| Small Heath | Full | Land-cover + Linear | 0.260 | 0.933 | 11845.4 |
| Small Heath | Full | Land-cover * Linear | 0.261 | 0.933 | 11848.2 |
| Small Heath | Agriculture | Land-cover | 0.127 | 0.934 | 11894.7 |
| Small Heath | Agriculture | Land-cover + Linear | 0.169 | 0.934 | 11874.5 |
| Small Heath | Agriculture | Land-cover * Linear | 0.170 | 0.933 | 11877.5 |
| Small Heath | Linear |  | 0.150 | 0.942 | 11926.8 |
| Small Heath | Year |  | 0.148 | 0.942 | 11935.0 |
| Orange Tip | Full | Land-cover | 0.112 | 0.727 | 10153.9 |
| Orange Tip | Full | Land-cover + Linear | 0.112 | 0.726 | 10155.8 |
| Orange Tip | Full | Land-cover * Linear | 0.114 | 0.726 | 10157.6 |
| Orange Tip | Agriculture | Land-cover | 0.067 | 0.726 | 10156.3 |
| Orange Tip | Agriculture | Land-cover + Linear | 0.073 | 0.724 | 10155.4 |
| Orange Tip | Agriculture | Land-cover * Linear | 0.074 | 0.724 | 10157.8 |
| Orange Tip | Linear |  | 0.072 | 0.735 | 10169.8 |
| Orange Tip | Year |  | 0.072 | 0.735 | 10167.6 |
| Brimstone | Full | Land-cover | 0.105 | 0.897 | 11696.1 |
| Brimstone | Full | Land-cover + Linear | 0.105 | 0.897 | 11699.8 |
| Brimstone | Full | Land-cover * Linear | 0.112 | 0.896 | 11699.2 |
| Brimstone | Agriculture | Land-cover | 0.024 | 0.901 | 11717.6 |
| Brimstone | Agriculture | Land-cover + Linear | 0.025 | 0.901 | 11720.9 |
| Brimstone | Agriculture | Land-cover * Linear | 0.043 | 0.900 | 11712.5 |
| Brimstone | Linear |  | 0.042 | 0.902 | 11714.3 |
| Brimstone | Year |  | 0.042 | 0.903 | 11710.8 |
| Small Copper | Full | Land-cover | 0.131 | 0.778 | 9743.3 |
| Small Copper | Full | Land-cover + Linear | 0.132 | 0.777 | 9743.9 |
| Small Copper | Full | Land-cover * Linear | 0.136 | 0.775 | 9743.8 |
| Small Copper | Agriculture | Land-cover | 0.071 | 0.778 | 9750.5 |
| Small Copper | Agriculture | Land-cover + Linear | 0.090 | 0.775 | 9741.6 |
| Small Copper | Agriculture | Land-cover * Linear | 0.098 | 0.774 | 9739.7 |
| Small Copper | Linear |  | 0.091 | 0.789 | 9750.1 |
| Small Copper | Year |  | 0.090 | 0.792 | 9752.9 |
| Meadow Brown | Full | Land-cover | 0.229 | 0.891 | 25872.1 |
| Meadow Brown | Full | Land-cover + Linear | 0.234 | 0.893 | 25870.6 |
| Meadow Brown | Full | Land-cover * Linear | 0.240 | 0.893 | 25871.8 |
| Meadow Brown | Agriculture | Land-cover | 0.143 | 0.894 | 25920.6 |
| Meadow Brown | Agriculture | Land-cover + Linear | 0.142 | 0.896 | 25919.6 |
| Meadow Brown | Agriculture | Land-cover * Linear | 0.152 | 0.894 | 25920.2 |
| Meadow Brown | Linear |  | 0.115 | 0.921 | 26007.5 |
| Meadow Brown | Year |  | 0.115 | 0.920 | 26004.6 |
| Gatekeeper / Hedge Brown | Full | Land-cover | 0.114 | 0.905 | 18478.7 |
| Gatekeeper / Hedge Brown | Full | Land-cover + Linear | 0.117 | 0.906 | 18478.7 |
| Gatekeeper / Hedge Brown | Full | Land-cover * Linear | 0.119 | 0.905 | 18479.4 |
| Gatekeeper / Hedge Brown | Agriculture | Land-cover | 0.062 | 0.903 | 18488.2 |
| Gatekeeper / Hedge Brown | Agriculture | Land-cover + Linear | 0.068 | 0.905 | 18487.3 |
| Gatekeeper / Hedge Brown | Agriculture | Land-cover * Linear | 0.071 | 0.903 | 18486.8 |
| Gatekeeper / Hedge Brown | Linear |  | 0.068 | 0.908 | 18497.3 |
| Gatekeeper / Hedge Brown | Year |  | 0.068 | 0.908 | 18496.1 |
| Marbled White | Full | Land-cover | 0.198 | 0.939 | 11120.0 |
| Marbled White | Full | Land-cover + Linear | 0.198 | 0.939 | 11122.3 |
| Marbled White | Full | Land-cover * Linear | 0.208 | 0.939 | 11122.8 |
| Marbled White | Agriculture | Land-cover | 0.077 | 0.941 | 11136.6 |
| Marbled White | Agriculture | Land-cover + Linear | 0.080 | 0.940 | 11139.2 |
| Marbled White | Agriculture | Land-cover * Linear | 0.087 | 0.942 | 11140.9 |
| Marbled White | Linear |  | 0.084 | 0.944 | 11149.5 |
| Marbled White | Year |  | 0.085 | 0.943 | 11150.6 |
| Ringlet | Full | Land-cover | 0.161 | 0.911 | 18798.1 |
| Ringlet | Full | Land-cover + Linear | 0.167 | 0.911 | 18797.7 |
| Ringlet | Full | Land-cover * Linear | 0.170 | 0.910 | 18799.3 |
| Ringlet | Agriculture | Land-cover | 0.076 | 0.908 | 18843.0 |
| Ringlet | Agriculture | Land-cover + Linear | 0.078 | 0.908 | 18843.4 |
| Ringlet | Agriculture | Land-cover * Linear | 0.088 | 0.908 | 18838.9 |
| Ringlet | Linear |  | 0.082 | 0.913 | 18858.8 |
| Ringlet | Year |  | 0.082 | 0.914 | 18858.9 |
| Peacock | Full | Land-cover | 0.111 | 0.750 | 16649.5 |
| Peacock | Full | Land-cover + Linear | 0.126 | 0.752 | 16640.5 |
| Peacock | Full | Land-cover * Linear | 0.125 | 0.751 | 16643.6 |
| Peacock | Agriculture | Land-cover | 0.073 | 0.753 | 16652.5 |
| Peacock | Agriculture | Land-cover + Linear | 0.083 | 0.753 | 16648.2 |
| Peacock | Agriculture | Land-cover * Linear | 0.092 | 0.751 | 16645.5 |
| Peacock | Linear |  | 0.088 | 0.764 | 16650.2 |
| Peacock | Year |  | 0.086 | 0.770 | 16660.4 |
| Large Skipper | Full | Land-cover | 0.107 | 0.779 | 13135.2 |
| Large Skipper | Full | Land-cover + Linear | 0.119 | 0.779 | 13128.8 |
| Large Skipper | Full | Land-cover * Linear | 0.118 | 0.778 | 13132.0 |
| Large Skipper | Agriculture | Land-cover | 0.032 | 0.783 | 13154.7 |
| Large Skipper | Agriculture | Land-cover + Linear | 0.039 | 0.782 | 13154.0 |
| Large Skipper | Agriculture | Land-cover * Linear | 0.043 | 0.781 | 13155.8 |
| Large Skipper | Linear |  | 0.042 | 0.787 | 13146.5 |
| Large Skipper | Year |  | 0.041 | 0.790 | 13147.0 |
| Speckled Wood | Full | Land-cover | 0.194 | 0.925 | 16342.4 |
| Speckled Wood | Full | Land-cover + Linear | 0.194 | 0.925 | 16346.3 |
| Speckled Wood | Full | Land-cover * Linear | 0.197 | 0.925 | 16349.6 |
| Speckled Wood | Agriculture | Land-cover | 0.140 | 0.926 | 16351.9 |
| Speckled Wood | Agriculture | Land-cover + Linear | 0.145 | 0.925 | 16353.0 |
| Speckled Wood | Agriculture | Land-cover * Linear | 0.150 | 0.925 | 16353.6 |
| Speckled Wood | Linear |  | 0.136 | 0.932 | 16401.7 |
| Speckled Wood | Year |  | 0.134 | 0.933 | 16402.6 |
| Wall Brown | Full | Land-cover | 0.299 | 0.835 | 3613.4 |
| Wall Brown | Full | Land-cover + Linear | 0.301 | 0.834 | 3613.5 |
| Wall Brown | Full | Land-cover * Linear | 0.347 | 0.843 | 3608.9 |
| Wall Brown | Agriculture | Land-cover | 0.056 | 0.825 | 3648.1 |
| Wall Brown | Agriculture | Land-cover + Linear | 0.062 | 0.826 | 3647.6 |
| Wall Brown | Agriculture | Land-cover * Linear | 0.064 | 0.826 | 3650.6 |
| Wall Brown | Linear |  | 0.063 | 0.830 | 3646.4 |
| Wall Brown | Year |  | 0.064 | 0.825 | 3647.6 |
| Large White | Full | Land-cover | 0.322 | 0.805 | 16314.3 |
| Large White | Full | Land-cover + Linear | 0.324 | 0.809 | 16312.7 |
| Large White | Full | Land-cover * Linear | 0.327 | 0.809 | 16312.6 |
| Large White | Agriculture | Land-cover | 0.299 | 0.808 | 16309.8 |
| Large White | Agriculture | Land-cover + Linear | 0.299 | 0.814 | 16309.4 |
| Large White | Agriculture | Land-cover * Linear | 0.302 | 0.812 | 16310.4 |
| Large White | Linear |  | 0.225 | 0.860 | 16427.9 |
| Large White | Year |  | 0.229 | 0.857 | 16433.7 |
| Green-veined White | Full | Land-cover | 0.191 | 0.825 | 16317.5 |
| Green-veined White | Full | Land-cover + Linear | 0.190 | 0.826 | 16320.3 |
| Green-veined White | Full | Land-cover * Linear | 0.194 | 0.825 | 16322.9 |
| Green-veined White | Agriculture | Land-cover | 0.111 | 0.830 | 16350.9 |
| Green-veined White | Agriculture | Land-cover + Linear | 0.112 | 0.829 | 16353.7 |
| Green-veined White | Agriculture | Land-cover * Linear | 0.120 | 0.831 | 16352.1 |
| Green-veined White | Linear |  | 0.114 | 0.838 | 16375.3 |
| Green-veined White | Year |  | 0.114 | 0.839 | 16372.0 |
