# Supplementary material for: A national‐scale model of linear features improves predictions of farmland biodiversity
Source: J Appl Ecol. 2017 May 7;54(6):1776–84. doi: 10.1111/1365-2664.12912 (PMC5697618; doi:10.1111/1365-2664.12912)
Supplement: Supplementary file 5 — Table S4. Effect of linear features variable type on abundance models for each species. [file JPE-54-1776-s005.docx]

**Table S4.** Effect of using total linear features length or woody linear features length on abundance model performance. For each species, the best performing model (lowest AIC) is shown in bold.

| Species | Best model | AIC with linear features term | AIC with woody linear features term |
| --- | --- | --- | --- |
| Buzzard | Full, additive | **10530.1** | 10541.7 |
| Corn Bunting | Agriculture, additive | **4162.1** | 4183.7 |
| Chaffinch | Full, interaction | 68213.2 | **68194.2** |
| Kestrel | Agriculture, additive | 8211.9 | **8144.6** |
| Lapwing | Full, interaction | **14409.4** | 14412.8 |
| Linnet | Full, interaction | 31918.0 | **31884.9** |
| Lesser Whitethroat | Full, additive | 7738.1 | **7726.2** |
| Grey Partridge | Linear only | **7356.6** | 7435.1 |
| Reed Bunting | Full, additive | 13583.7 | **13567.1** |
| Rook | Agriculture, additive | 34159.2 | **34055.1** |
| Skylark | Full, additive | 39764.2 | **39757.6** |
| Stock Dove | Agriculture, additive | **14601.3** | 14668.7 |
| Barn Swallow | Agriculture, additive | **31278.1** | 31294.9 |
| Turtle Dove | Agriculture, additive | **3438.8** | 3468.6 |
| Tree Sparrow | Agriculture, additive | **6770.3** | 6782.3 |
| Common Whitethroat | Full, interaction | 65348.3 | **65347.7** |
| Yellowhammer | Full, interaction | **30345.8** | 30392.6 |
| Yellow Wagtail | Agriculture, interaction | **3983.9** | 4005.6 |
| Small White | Full, additive | **16811.2** | 16812.1 |
| Comma | Full, additive | 11471.2 | **11470.7** |
| Common Blue | Full, additive | 16516.6 | **16504.4** |
| White-letter Hairstreak | Linear only | **598.6** | 603.4 |
| Purple Hairstreak | Linear only | 2692.9 | **2692.0** |
| Essex Skipper | Linear only | **2760.5** | 2767.2 |
| Small Skipper | Full, additive | 10301.4 | **10299.5** |
| Small Tortoiseshell | Full, additive | 11372.4 | **11369.0** |
| Brown Argus | Agriculture, additive | 6791.8 | **6773.5** |
| Holly Blue | Linear only | **6232.6** | 6233.0 |
| Small Heath | Full, additive | 11845.4 | **11830.0** |
| Orange Tip | Agriculture, additive | 10155.4 | **10145.8** |
| Brimstone | Full, interaction | 11699.2 | **11693.8** |
| Small Copper | Agriculture, interaction | 9739.7 | **9738.6** |
| Meadow Brown | Full, additive | **25870.6** | 25875.5 |
| Gatekeeper / Hedge Brown | Full, additive | **18478.7** | 18482.5 |
| Marbled White | Full, additive | 11122.3 | **11114.3** |
| Ringlet | Full, additive | **18797.7** | 18799.9 |
| Peacock | Full, additive | 16640.5 | **16640.4** |
| Large Skipper | Full, additive | **13128.8** | 13138.7 |
| Speckled Wood | Full, additive | 16346.3 | **16343.0** |
| Wall Brown | Full, interaction | 3608.9 | **3601.0** |
| Large White | Agriculture, additive | 16309.4 | **16308.5** |
| Green-veined White | Full, additive | 16320.3 | **16320.1** |
